# Fuzziness of muscle synergies in patients with multiple sclerosis indicates increased robustness of motor control during walking

**DOI:** 10.1038/s41598-020-63788-w

**Published:** 2020-04-29

**Authors:** Lars Janshen, Alessandro Santuz, Antonis Ekizos, Adamantios Arampatzis

**Affiliations:** 1 Department of Training and Movement Sciences, Humboldt-Universität zu Berlin, Philippstraße 13, Berlin, 10115 Germany; 2 Berlin School of Movement Science, Humboldt-Universität zu Berlin, Philippstraße 13, Berlin, 10115 Germany

**Keywords:** Motor control, Multiple sclerosis

## Abstract

Deficits during gait poses a significant threat to the quality of life in patients with Multiple Sclerosis (MS). Using the muscle synergy concept, we investigated the modular organization of the neuromuscular control during walking in MS patients compared to healthy participants (HP). We hypothesized a widening and increased fuzziness of motor primitives (e.g. increased overlap intervals) in MS patients compared to HP allowing the motor system to increase robustness during walking. We analysed temporal gait parameters, local dynamic stability and muscle synergies from myoelectric signals of 13 ipsilateral leg muscles using non-negative matrix factorization. Compared to HP, MS patients showed a significant decrease in the local dynamic stability of walking during both, preferred and fixed (0.7 m/s) speed. MS patients demonstrated changes in time-dependent activation patterns (motor primitives) and alterations of the relative muscle contribution to the specific synergies (motor modules). We specifically found a widening in three out of four motor primitives during preferred speed and in two out of four during fixed speed in MS patients compared to HP. The widening increased the fuzziness of motor control in MS patients, which allows the motor system to increase its robustness when coping with pathology-related motor deficits during walking.

## Introduction

Multiple Sclerosis (MS) is a chronic inflammatory-mediated degenerative neurological disease resulting in demyelination of the central nervous system (CNS)^1,2^. The heterogeneous pathological and clinical presentation of MS may result in deficits in motor, sensory as well as cognitive function^3^. The predominantly progressive disease is affecting more than 2.3 million people worldwide with high incidence in young adults^4,5^. The increased relevance of the disease is supported by a recent study, reporting a remarkably high prevalence of more than 900.000 patients only in the US^6^. Approximately 75% of MS patients experience clinically relevant walking disturbances^7,8^. These include a reduced walking speed, lower cadence and shorter step length^9–11^. Up to 85% of MS patients report mobility impairments^12^, and gait is perceived as the most important bodily function across the MS disability spectrum^13^. Moreover, studies reported that more than 50% of MS patients fall within six months^14^ or one year^15^ with a three times higher risk of falls for females with MS compared to healthy age matched controls^16^. More than 45% of falls resulted from external perturbations such as slipping or tripping^17^. The highest risk of multiple falls in middle-aged patients with MS have been reported for persons with mobility impairments who did not use an assistive device^18^.

During perturbed locomotion the motor system has to increase robustness, which is the ability to cope with perturbations^19,20^ in order to maintain stability and to avoid falls. In healthy participants Santuz, *et al*.^20^ observed an increased robustness of the motor system during walking and running in the presence of continuous external perturbations induced by an uneven surface. In MS patients the pathology related degeneration of the neuro-muscular system and the resulted deficits in the motor control efficiency can be presented as perturbations generated from internal, physiological sources^21^. To what extent MS patients might modify their motor control output to cope with these internal, pathology related perturbations during daily life locomotion is not well understood. It has been reported that MS patients show higher activation levels in the premotor cortex suggesting an increased challenge during walking compared to healthy controls^22^. In healthy participants an increased challenge during walking and running resulting from external, environmental sources has been associated with an increased robustness in motor control^20^. We expect a similar increased robustness in motor output in MS patients to cope with the internal, pathology related perturbations compared to healthy participants.

Walking as a dynamic task requires maintaining a high level of movement complexity and safety while task variability is induced from the environment. This task is accomplished by muscle coordination, controlling the activation and timing of multiple muscles^23^. A common hypothesis in motor control is that the CNS may simplify movement generation by activating muscles in common patterns called muscle synergies^24,25^. The created motor output are based on the interplay of spinal and supraspinal interactions with the environment^26^. Depending on the motor task and the environmental conditions the activation of specific muscles is grouped together into muscle synergies that consist of a time-dependent component called “motor primitives”^27^ and the time-invariant muscle weights, referred to as “motor modules”^28^. In this way synergies provide a small set of commands, that can be combined to obtain complex movements^29^. In the lower limb of healthy participants (HP) four muscle synergies have been found to sufficiently describe the walking task^20,30,31^. A similar number of synergies during walking was observed in patients with cerebellar ataxia^32^ and MS^33^. Comparing MS patients with healthy controls Lencioni and colleagues^33^ described alterations in the modular control dominated by modifications of the activation timing rather than module composition. However, the used analysis methods of Lencioni did not allow for the identification of a widening or overlap of the motor primitives. Assuming a neural origin of muscle synergies, internal pathology-related perturbations might alter the modular organization of the neuromuscular system. In this terms MS may provide an adequate model to investigate mechanisms of the neuromuscular system to cope with pathology induced internal perturbations.

The purpose of the current study was to investigate the modular organization in MS patients during walking and to identify adjustments in motor control output associated with the neuromuscular weakness of these patients. We hypothesized a widening of the motor primitives in MS patients to compensate for pathology-related internal perturbations as a strategy to increase the robustness of the motor system during walking.

## Materials and Methods

### Participants

Twenty female participants divided into two subgroups volunteered in the experiments. The HP group consisted of 10 healthy participants (age: 56 ± 4 years, body mass: 68 ± 16 kg, body height: 1.73 ± 0.07 m, BMI: 23.0 ± 3.3 kg/m^2^) who were physically active, did not use orthotic insoles and had no known history of neurological or motor disorders or injuries over the six months prior to the measurements. Regarding leg laterality, in this control group six participants were right- and four left-dominant. The criteria for the MS patients to participate in the study included the age of 45–70 years, the ability to walk a minimum of 500 m without assistance or assistive devices and a common experience in treadmill walking. In the group, consisting of 10 MS patients (age: 55 ± 8 years, body weight: 65 ± 9 kg, body height: 1.68 ± 0.05 m, BMI: 23.0 ± 2.5 kg/m^2^), in five patients the left leg and in the other five the right leg was more affected. The patients were diagnosed for MS since 8 ± 2 years, all as relapsing remitting MS. At the time of measurements, they had no strike within the last three months and the averaged Expanded Disability Status Scale (EDSS) was 3.0 ± 1.0. For ethical reasons patients did not lower or interrupt their continuous medication for the measurements. All patients were able to continuously walk more than 500 m without assistance or assistive devices. On a scale from 1 (never) to 5 (always) they reported to have “rarely” (2 ± 1) difficulties in walking inside or outside their homes, respectively. Experiencing general gait or balance problems was in average classified as “occasionally” (3 ± 1). All participants of both groups had common experience in treadmill walking due to their physical activity or their rehabilitation procedures. This study was reviewed and approved by the Ethics Committee of the Humboldt-Universität zu Berlin (HU-KSBF-EK-2017003). All the participants gave written informed consent for the experimental procedure, in accordance with the Declaration of Helsinki.

#### Experimental design

To obtain the individual preferred walking speed for each participant, we used the method of limits^34^ performed on a treadmill (mercury, H-p-cosmos Sports & Medical GmbH, Germany). Starting at 0.8 m/s the speed was randomly increased by 0.02 to 0.05 m/s at varying time intervals of five to ten seconds. After the participant confirmed the preferred speed, the procedure was repeated by decreasing the speed starting from a pace of 0.5 to 1.0 m/s higher than the preferred speed. The preferred speed was calculated by the average speed of both tests. If the difference between tests was larger than 10%, the test was repeated. Thereafter all participants randomly performed two trials (120 s each) at two speeds in level walking on the treadmill. The different speeds were the individual preferred speed and a fixed speed of 0.7 m/s. Resting-time between trials was 5 minutes. The measurements of the MS patients were scheduled in their self-reported individual high-performance time within the day. In each trial, the recordings began after a preliminary habituation phase in treadmill walking of around 30 s.

#### Gait cycle assessment

During walking plantar pressure distributions were measured at 120 Hz by a pressure plate (FDM-THM-S, zebris Medical GmbH, Germany) integrated in the treadmill. The pressure plate data were acquired synchronously to the EMG recording using the proprietary software (WinFDM-T v2.5.1, zebris Medical GmbH, Isny im Allgäu, Germany) and then extracted in a raw format to obtain gait cycles using a validated custom algorithm^35^. The pressure plate was synchronized with the EMG data by an analogue signal. In addition to the contact times, the pressure data were used to quantify step lengths, cadence, vertical ground reaction forces (VGRFs) and total impulse of the stance phase for the gait cycles. Forces and impulses were then normalized to body weight [BW]. The data of 30 consecutive gait cycles within each of the four recorded trials per participant were averaged.

#### Muscle activity assessment

The activity of 13 ipsilateral hip and leg muscles was recorded by surface EMG. We measured the dominant leg (5 × right and 5 × left leg) of the HP and more affected leg (4 × right and 3 × left leg) of the MS patients. The pairs of Ag/AgCl electrodes (N-00-S, Ambu, Denmark) for bipolar derivation were applied according to the SENIAM standards^36^. Signals were recorded at 1000 Hz and 16-bit resolution using a wireless EMG system (myon AG; Switzerland). The EMG system had a built-in band-pass filter (5–500 Hz, 3 dB/oct, 4th order). The recorded hip and upper leg muscles included gluteus medius (ME), maximus (MA) and tensor fasciae latae (TF) as well as rectus femoris (RF), vastus medialis (VM) and lateralis (VL), semitendinosus (ST) and the long head of the biceps femoris (BF). Lower leg muscles were tibialis anterior (TA), peronaeus longus (PL), gastrocnemius medialis (GM) and lateralis (GL) and soleus (SO). During the walking tasks 30 consecutive gait cycles were measured. EMG signals were high-pass filtered, full-wave rectified, and low-pass filtered at cut-off frequencies of 50 and 20 Hz respectively using 4^th^ order zero-lag IIR Butterworth filters according to Santuz, *et al*.^37^. The amplitude was normalized to the maximum activation of each muscles recorded for each participant at each condition^38,39^. Each EMG envelope was time-normalized to 200 data points per gait cycle. Each gait cycle was divided in 100 data points for the stance and 100 data points for the swing phase. There are two reasons for this choice^39^. First, dividing the gait cycle into two macro-phases helps to understand the temporal contribution of the different synergies, diversifying between stance and swing. Second, normalizing the duration of stance and swing to the same number of points for all participants (and for all the recorded gait cycles of each participant) makes the interpretation of the results independent from the absolute duration of the gait events.

#### Spinal motor output assessment

For the indirect spinal motor output characterization, we mapped the EMG activity onto the estimated rostrocaudal location of alpha-motoneurons (MNs) pools in the segments from the second lumbar vertebra (L2) to the second sacral vertebra (S2) of the spinal cord^31,40,41^. The contribution of each muscle to the total estimated activity of the spinal segments was implemented using the myotomal charts developed by^41^, Kendall^42^. This method represents the organization of the efferent MNs network, assuming a common spinal topography among the participants included in this study. The motor output of each spinal segment S_j_ was estimated using the Eq. (1) introduced by La Scaleia, *et al*.^41^:

1 $${S}_{j}=\,\frac{{\sum }_{i=1}^{{m}_{j}}\left(\frac{{k}_{ji}}{{n}_{i}}\times EM{G}_{i}\right)}{{\sum }_{i=1}^{{m}_{j}}\left(\frac{{k}_{ji}}{{n}_{i}}\right)}\times M{N}_{j}$$

where *m*_*j*_ are the muscles innervated by each segment, *n*_*i*_ is the number of spinal levels that innervate the *i*^th^ muscle k_ji_ is a weighting coefficient specific to each muscle and spinal segment (e.g. *k*_*ji*_ = 1 or *k*_*ji*_ = 0.5 if *S* is a major or minor MN source, respectively) and EMG_i_ is the normalized recorded EMG, specific for each participant and trial^41,42^. This approach account for size differences at each spinal level in every *MN*_*j*_ pool.

#### Modular organization assessment

Muscle synergy extraction was performed by applying a customized script^37^ (R v3.4.4, R Found. for Stat. Comp.) using the classical Gaussian non-negative matrix factorization (NMF) algorithm^43,44^. The original data matrix (V_13×6000_) consisted of 13 rows (number of muscles) and 6000 columns (200 data points in time per normalized gait cycle from 30 concatenated cycles). To determine motor primitives and the respective motor modules of the muscle synergies across all EMG signals, the original matrix was factorized into two smaller matrices. The matrix (H) representing the motor primitive consists of N rows and 6000 columns, where N is the number of synergies. The matrix (W) consisting of 13 rows and N columns represents the motor modules. The combination of H and W describes the synergies necessary to accomplish a walking trial. The reconstruction of the original EMG matrix was performed by multiplying the motor module with the motor primitive matrices (V_R_ = W_13xN_ ·H_Nx6000_). The reconstruction quality was assessed by the coefficient of determination (R^2^) between the original and the reconstructed matrix^45^ calculated as described by Santuz, *et al*.^37^.

The limit of convergence was reached when a change in the calculated R^2^ between V and V_R_ was smaller than 0.01% in the last 20 iterations^45^, meaning that, with that amount of synergies, the signal could not be reconstructed any better. This operation was first completed by setting the number of synergies to 1. Then, it was repeated by increasing the number of synergies each time, until a maximum of 10 synergies. The number 10 was chosen to be lower than the number of muscles, since extracting a number of synergies equal to the number of measured EMG activities would not reduce the dimensionality of the data. Specifically, 10 is the rounded 75% of 13, which is the number of considered muscles. The computation was repeated 10 times for each synergy, each time creating new randomized initial matrices, in order to avoid local minima^46^. These results were plotted as a curve of R^2^ vs. the number of synergies. To determine a sufficient number of synergies required to represent the original EMG signals, we applied a method based on the cross-validation of the R^2^ values obtained for each number of synergies^45^. The curve of R^2^ vs. the number of synergies was fitted by a simple linear regression model that initially uses all the ten synergies. The mean squared error between the curve and the linear interpolation was then calculated. Afterwards, the first point in the R^2^-vs.-synergies curve was removed and the error between this new curve and its new linear interpolation was calculated. This procedure was repeated until only two points were left on the curve or until the mean squared error fell below 10^−5^ ^45^. This method searches for the most linear part of the R^2^-vs.-synergies curve and it is equivalent to stating that the reconstruction quality is not improving much when the curve becomes linear. With this approach, the need for setting a threshold to the reconstruction quality is avoided, giving space to the possibility that quality might not improve at the same rate when the same NMF algorithm is applied to different data. The procedure described above resulted in a specific number of synergies for each of the total 80 trials (20 participants, 4 trial per participant) necessary to sufficiently reconstruct the respective original EMG dataset. After the factorization and reconstruction procedure the concatenated motor primitives were split into the single gait cycles.

In some cases, the factorization produces synergies that can be modelled from a combination of two or more simpler synergies. According to our previous work, we classified fundamental and combined synergies, where fundamental synergies were characterized by motor primitives that demonstrated a single major activation peak^37,47,48^. A combined synergy is characterized by more than one activation peak in the motor primitives and a combination of the respective associated muscle contributions in the motor modules. The synergy recognition was performed using an established procedure that consists of a combination of visual inspection and automated iterative recognition based on a curve-fitting-model^20,37,47,48^. In our data, combined synergies occur in about 20% of the total extracted synergies. This level of occurrence was consistent across participant groups and walking velocities.

#### Metrics for comparison of curves

To increase the information value of the measured parameters by taking into account potential variations within participants, we included both trials of each walking speed of each participant. We evaluated the similarities of the motor primitives between the healthy participants and the MS patients by calculating the coefficient of determinations (R^2^) defined as

2 $${R}^{2}=1-\frac{{({H}_{1}-{H}_{2})}^{2}}{{({H}_{1}-\overline{{H}_{1}})}^{2}}$$

for any two metrics of *H*_*1*_ and *H*_*2*_ of equal dimensions to be compared. The following steps were applied for each of the respective motor primitives. In the first step we calculated the averaged similarity between the first trial of a participant and all trials of all participants of the same group (e.g. HP and MS patients, respectively). Then we perform the same with the second trial and averaged both results. Repeating this procedure for all participants resulted in ten within group similarity values for each group (e.g. w HP and w MSP) for each of the walking speeds, separately. In the next step we performed the same procedure separately calculating the averaged similarity between the first, then the second trial of one HP to all trials of all MS patients. Averaging the values for each HP resulted in ten between group similarities for HP vs. MS patients.

For the activation of the extracted spinal motor output and the resulting curves of the motor primitives (matrix H) we characterized the activation duration and timing by evaluating the full width at half maximum (FWHM) and the centre of activity (CoA), respectively. The FWHM was calculated as the number of points exceeding half of the curve’s maximum, after subtracting the minimum of the gait cycle^49^. For each time interval relative to gait cycle, we calculate the frequency of the overlapping intervals of any motor primitive. An overlap occurred when at least two primitives were exceeding half maximum. The normalisation, FWHM, CoA, and overlaps were calculated per step and then averaged for the respective participant and walking condition. Using circular statistics^49^, the CoA was defined as the centre of mass of the motor primitive projected to the time domain of the gait cycle. For every trial, FWHM and CoA were calculated for each gait cycle and then averaged over the 30 gait cycles to proceed with the statistical analysis. For the spinal motor outputs, both parameters were analysed and time scaled for stance and swing distinctively. For the motor primitives of the muscle synergies FWHM and CoA were analysed and time scaled over the whole gait cycle. This is based on the concept, that muscle synergies and therefore motor primitives are stance or swing specific, while the spinal motor output is not.

#### Local dynamic stability assessment

The local dynamic stability represents the ability of the system to respond to small internal perturbations^50^, representing how the system is able to maintain stability and detects neuromuscular control errors in achieving it^51,52^. To assess the local dynamic stability, we adopted the maximum finite-time Lyapunov exponents (MLE) of the human system during walking. The accelerations measured by wireless 3D-accelerometers (myon AG; Switzerland) placed at the second lumbar vertebrae (L2) as this bone landmark exhibits high reliable MLE values^53^ and approximates the centre of mass during walking. For all participant was recorded at 2000 Hz for the duration of 90 s in both trials of the two walking speeds. The captured data were low-pass filtered with a 4^th^ order zero-lag IIR Butterworth filter at cut-off frequencies of 15 Hz and were down sampled by a factor of 10. We calculated the MLE using the norm of the acceleration in all three axes. To avoid dependencies on step frequency, we identified the maximum number of shared steps (i.e. 0.5 of gait cycle) for all trials and participants^50^ resulting in 110 steps for walking at preferred speed and 100 steps at fixed speed (0.7 m/s), respectively. The acceleration data segments corresponding to the exact number of steps were then isolated for each trial. Following, the data segments were normalized to a uniform length amounting to ~18000 data points per trial. State space reconstruction was achieved through delay coordinate embedding^54,55^, for each point of the time series and its time-delayed copies as follows (Eq. 2):

3 $$S(t)=[z(t),\,z(t\,+\tau ),\,\ldots ,\,z(t+(m-1)\tau )]$$

with *S(t)* being the m-dimensional reconstructed state vector, *z(t)* the input 1D coordinate series, τ the time delay and *m* the embedding dimension. Time delays were calculated for each time series from the first minimum of the mutual-information curve, based on the Average Mutual Information function^56^.

For our data, an embedding dimension of 3 was sufficient for the state-space reconstructions. Different values of *τ* can yield very different state-space reconstructions^57–59^. It is therefore suggested that individualized values of *τ* are best to represent a dynamical system^53,60^. However, due to the independence of the two groups a common delay was utilized for the reconstruction of all signals excluding any effect of delay from the between group comparison. Time delays were selected based on the average of all trials and participants and ranged from 33 to 35 data points for preferred and fixed speed, respectively (~0.2 of the average step time).

Following the reconstruction of the times series, the Rosenstein algorithm was used to compute the average exponential rate of divergence by calculating the Euclidean distance of each point’s trajectory divergence to its closest trajectory^51,61^. The MLEs were then calculated as the slope of the linear fit in the resulting divergence curves from 0 to 0.25 of the delay. Data analysis was performed on MATLAB 2014b (Mathworks Inc., Natick, United States).

#### Statistical analysis

In the statistical analysis we used a t-test or Wilcoxon-Mann-Whitney test to evaluate differences between MS patients and the control group for the two walking speeds separately. The analysis was performed for the temporal and spatial walking parameters, MLE values, R^2^ similarities (e.g. comparing the within-group similarities as well as each of the within-group similarities to the between-group similarity), the FWHM, and the CoA of the motor primitives. According to the purpose of this study, we focus our attention on parameters of all muscle synergies that can be paired across the two participant groups of HP and MSP (e.g. the fundamental synergies). The statistical power was calculated for a two samples, two sided comparisons of means^62^. For the analysis of the projected spinal motor output a two-way ANOVA with the factors participant and spinal segment followed by a Tukey post-hoc analysis was used. A two-way ANOVA for repeated measures with the factors participant group (HP and MS patients) and the muscles followed by a Tukey post-hoc analysis with false discovery rate p-value adjustment was used to evaluate differences in the motor modules separately for both walking speeds. All the significance levels were set to α = 0.05 and the statistical analyses were conducted using R v3.4.4 (R Found. for Stat. Comp.).

## Results

### Gait parameters

The MS patients demonstrated a significantly (p = 0.002, power = 0.96) lower preferred speed of 0.8 ± 0.4 m/s (HP = 1.3 ± 0.1 m/s) and a significantly shorter (p = 0.001, power = 0.66) step length (0.70 ± 0.21 m) compared to HP (0.86 ± 0.04 m). In addition, the cadence in MS patients was significantly (p = 0.003, power = 0.93) lower (97 ± 13 steps/min) than in HP (113 ± 7 steps/min) and the gait cycle time of MS patients (1.20 ± 0.12 s) was significantly (p < 0.001, power = 0.98) longer than in HP (1.06 ± 0.06 s). The contact time and the double stance time normalized to gait cycle were significantly longer (both p < 0.001, power = 0.87 and 0.89, respectively) in MS patients (73 ± 7% and 20 ± 5% of the gait cycle, respectively) compared to the HP (66 ± 2% and 15 ± 2% gait cycle, respectively). In the fixed walking speed condition, no significant differences were observed for step length (0.50 ± 0.05 m to 0.50 ± 0.06 m), cadence (83 ± 9 steps/min to 85 ± 9 steps/min), gait cycle times (1.40 ± 0.12 s to 1.39 ± 0.14 s), contact times (70 ± 3% to 70+2% of gait cycle) and double stance time (19 ± 1% to 18 ± 2% of gait cycle) between MS patients and HP, respectively.

### Local dynamic stability

**Figure 1:**
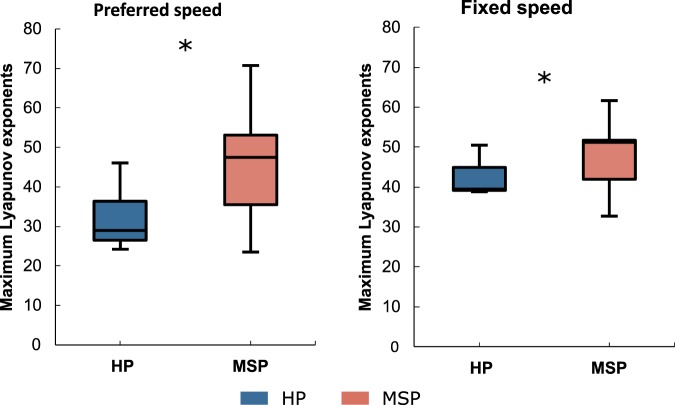
Boxplots depicting the maximum Lyapunov exponent values for healthy participants (HP) and patients with Multiple Sclerosis (MSP) during walking at preferred and fixed speed. Asterisks denote significant differences between HP and MSP.

The MLE were significantly (p < 0.001, power = 0.99) higher in MS patients compared to HP for both walking speeds (Fig. 1) evidencing a higher instability in the MS patients during walking.

### Spinal motor output

**Figure 2:**
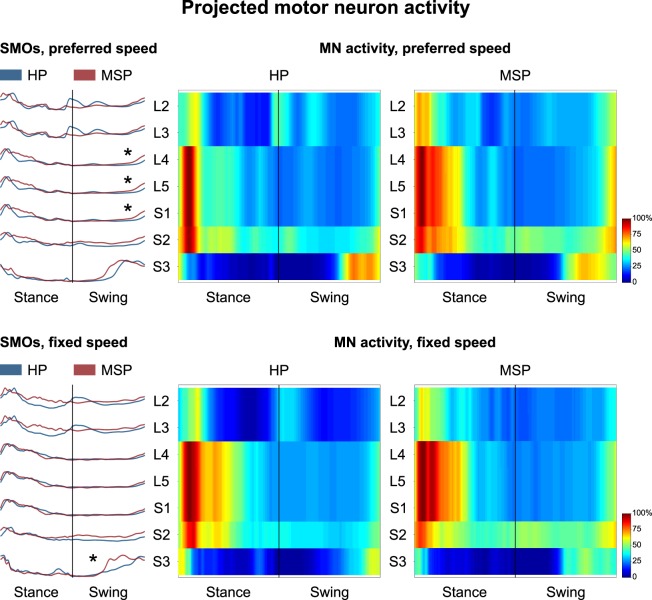
The average spatiotemporal spinal motor outputs (SMOs) are presented for healthy participants (HP) and patients with Multiple Sclerosis (MSP) during walking at preferred and fixed speed normalized in amplitude to the maximum of each segment. These curves were obtained by mapping each of the 13 muscle activations onto the relevant spinal segment (lumbar from L2 to L5 and sacral from S1 and S3). Asterisks denote significant differences in the full width at half maximum of the mapped EMGs between HP and MSP. The two level plots show the average alpha-motoneurons (MN) activity for each condition, giving additional information about the absolute activation level (normalization to the maximum of each condition). The stance and swing phases have been temporally normalized to the same amount of data points (100 each). Values are the means across all participants and all trials.

For the preferred and fixed walking velocity the two-way-ANOVA reviled the factors participant group and spinal segment as the significant (all p < 0.001). During walking at preferred speed, the averaged spatiotemporal spinal motor output for the segments L4, L5 and S1 projected from the measured EMG data demonstrated a significantly (p = 0.049, power = 0.97) larger FWHM in MS patients (15.8 ± 4.1% gait cycle) compared to HP (12.2 ± 3.3% gait cycle) (Fig. 2). In the fixed speed walking condition significantly (p = 0.012, power = 0.93) larger FWHM (13.4 ± 2.9% gait cycle) were observed in MS patients compared to HP (8.5 ± 3.8% gait cycle) for the segment S3.

### Modular organization

**Figure 3:**
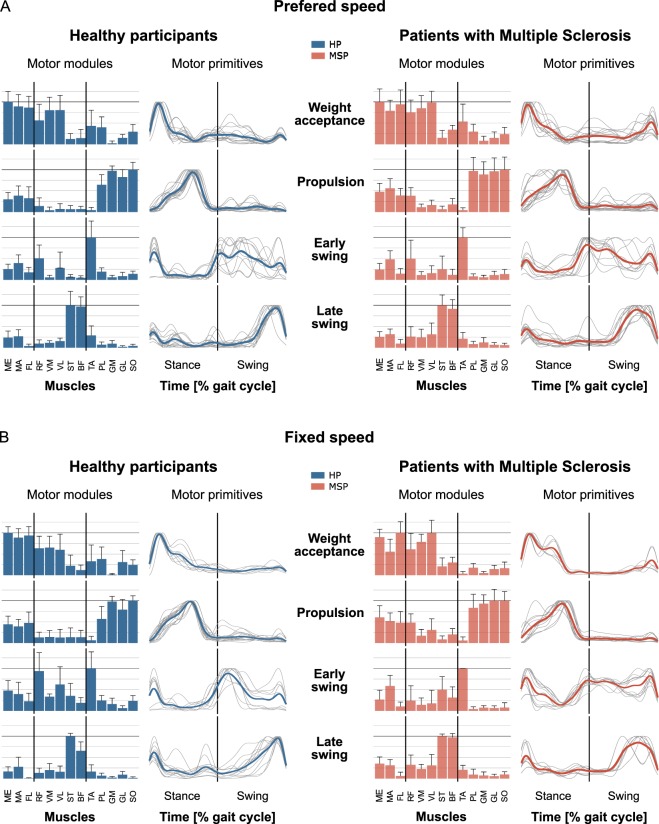
Averages and standard deviations of the motor modules as well as averaged and individual motor primitives of the four fundamental synergies for healthy participants (HP) and patients with Multiple Sclerosis (MSP) during walking at preferred speed (A, HP = 1.3 ± 0.1 m/s, MSP = 0.8 ± 0.4 m/s) and fixed speed (B, 0.7 m/s). The motor modules are presented on a normalized y-axis base (horizontal black line indicates normalized to 1). Included muscles are gluteus medius (ME), maximus (MA), tensor fasciae latae (TF), rectus femoris (RF), vastus medialis (VM), lateralis (VL), semitendinosus (ST), long head of biceps femoris (BF), tibialis anterior (TA), peronaeus longus (PL), medial (GM) and lateral (GL) gastrocnemius and soleus (SO). For the motor primitives, the x-axis full scale represents one gait cycle (stance and swing normalized to the same amount of points and divided by a vertical line) and the y-axis the normalized maximum amplitude of the individual motor primitives.

The minimum number of synergies necessary to sufficiently reconstruct the recorded EMG activity (i.e. the factorisation rank obtained through NMF) was not significantly different between HP and MS patients in either walking at preferred speed (HP = 3.8 ± 0.5, MS patients = 3.6 ± 0.5, p = 0.269) or walking at fixed speed (HP = 3.5 ± 0.6, MS patients = 3.5 ± 0.5, p = 0.980). The identified fundamental synergies extracted during walking were associated with different phases of the gait cycle (Fig. 3). The first synergy (peak at ~13% of the stance phase in HP and MS patients) functionally referred to body weight acceptance in the early stance phase. As shown by the motor modules, the first synergy mainly represented the activation of the hip extensors (ME, MA) and knee extensors (VM, VL, RF). The second synergy (peak at ~60% of the stance phase in HP and MS patients) describes the propulsion phase with large contribution of the plantar flexor muscles (GM, GL, SO and PL). The third synergy appeared during the transition from stance to swing. In the HP it ranged from early swing until the mid-swing phase (peak at ~15% of the swing phase) for both walking velocities. In MS patients it appeared slightly earlier and ranged from late stance until the late swing phase with a peak at ~lift-off and ~95% of the swing phase for preferred and fixed speed, respectively. In both participant groups this synergy predominantly represented the dorsi flexors (TA) as well as the RF in the function as hip flexor in the HP and the hip extensors (MA) and the knee flexor (ST) in the MS patients group. The fourth synergy (peak at ~88% of the swing phase in HP and 71% of the swing phase in MS patients) was related to the late swing phase including the preparation of the next foot contact and represented the activation of the knee flexors (ST, BFL).

**Figure 4:**
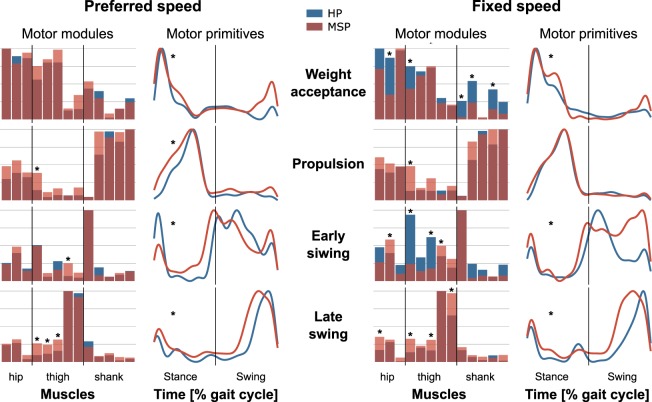
Average motor modules and motor primitives of the four fundamental synergies for walking at preferred and fixed speed. The motor modules are presented on a normalized y-axis base. Included muscles at the hip were gluteus medius (ME), maximus (MA) and tensor fasciae latae (TF), at the thigh rectus femoris (RF), vastus medialis (VM) and lateralis (VL), semitendinosus (ST) and the long head of biceps femoris (BF). Lower-leg muscles were tibialis anterior (TA), peronaeus longus (PL), medial (GM) and lateral (GL) gastrocnemius and soleus (SO). For the motor primitives, the x-axis full scale represents one gait cycle (stance and swing normalized to the same amount of points and divided by a vertical line) and the y-axis the normalized amplitude. Asterisks denote significant differences between healthy participants (HP) and patients with Multiple Sclerosis (MSP) for Post hoc tests in the motor modules and R^2^ similarities within groups compared to between groups in the motor primitives.

Within both participant groups no significant differences were observed for the R^2^ similarities of the motor primitives between both trials of the same walking speed. Corresponding to the variations in the individual motor primitives, the early swing synergy demonstrated the lowest similarities independent of the participant group and the walking condition. However, the R^2^ similarity within MS patients was lower for all synergies in both speeds compared to the HP. This phenomenon was significant for the motor primitives of the weight acceptance, propulsion and late swing during preferred speed, as well as for the motor primitives of the propulsion, early and late swing for the fixed speed (Table 1). Moreover, the R^2^ similarities within the respective groups of HP and MS patients were higher compared to the between-group similarities of the respective primitives at the same speeds. In the HP group the within-group R^2^ similarities were significant higher compared to the R^2^ similarities between the groups for all motor primitives except the propulsion during fixed speed walking. For MS patients all but the propulsion motor primitives in both speeds showed significant lower between-group similarities compared to the within-group values. (Fig. 4 and Table 1).

**Table 1:** Similarities of motor primitives, indicated as R^2^ values (average ± standard deviation) within the groups of healthy participants (w HP) and patients with Multiple Sclerosis (w MSP) as well as for the comparison of between groups (Btw Groups).

Speed	Muscle Synergy	w HP (R^2^)	w MSP (R^2^)	w HP vs. w MSP	Btw Groups (R^2^)	w HP vs. Btw Groups	w MSP vs. Btw Groups
				*p-value*		*p-value*	*p-value*
Preferred	Weight acceptance	0.530 ± 0.147	0.300 ± 0.148	0.003*	0.083 ± 0.138	0.001*	<0.003*
	Propulsion	0.662 ± 0.087	0.358 ± 0.117	<0.001*	0.392 ± 0.115	0.001*	<0.520
	Early swing	0.130 ± 0.541	0.137 ± 0.306	0.481	−0.583 ± 0.311	<0.003*	0.001*
	Late swing	0.657 ± 0.054	0.410 ± 0.107	<0.001*	0.257 ± 0.105	0.001*	<0.005*
Fixed	Weight acceptance	0.736 ± 0.090	0.810 ± 0.080	0.075	0.534 ± 0.090	<0.001*	<0.001*
	Propulsion	0.554 ± 0.078	0.489 ± 0.069	0.036*	0.450 ± 0.083	0.218	0.264
	Early swing	0.401 ± 0.306	0.234 ± 0.156	0.019*	−0.524 ± 0.341	<0.001*	<0.001*
	Late swing	0.696 ± 0.158	0.337 ± 0.174	0.001*	−0.046 ± 0.135	<0.001*	<0.001*

R^2^ values are given for walking at preferred speed (HP = 1.3 ± 0.1 m/s, MS patients = 0.8 ± 0.4 m/s) and fixed speed (0.7 m/s). The p-values were calculated to evaluate (1) if the R^2^ values within groups were different (w HP vs. w MSP), (2) if the within MS patients R^2^ differ from the Btw Groups R^2^ values (w MSP vs. Btw Groups) and (3) if the within HP R^2^ differ from the Btw Groups R^2^ values (w HP vs. Btw Groups).

At preferred walking speed, MS patients demonstrated a significantly increased FWHM of motor primitives in the weight acceptance, early and late swing synergies compared to the HP group. During walking at fixed speed, MS patients again showed significantly increased FWMH values in the weight acceptance and early swing motor primitives (Table 2).

**Table 2:** Full width at half maximum (FWHM, average values ± standard deviations in % of gait cycle) of the motor primitives for the healthy participants (HP) and patients with Multiple Sclerosis (MSP) walking at preferred speed (HP = 1.3 ± 0.1 m/s, MSP = 0.8 ± 0.4 m/s) and fixed speed (0.7 m/s).

Speed	Muscle Synergies	HP (%)	MSP (%)	*p-value*	*power*
**Full Width at Half Maximum (FWHM)**					
Preferred	Weight acceptance	9.3 ± 1.5	14.0 ± 4.2	0.006*	0.92
	Propulsion	15.9 ± 2.7	17.8 ± 3.7	0.219	0.25
	Early swing	26.3 ± 9.9	36.2 ± 8.6	0.029*	0.66
	Late swing	19.3 ± 1.7	23.5 ± 5.3	0.037*	0.65
Fixed	Weight acceptance	12.5 ± 1.4	18.9 ± 3.1	<0.001*	0.99
	Propulsion	18.4 ± 4.5	17.9 ± 5.7	0.740	0.06
	Early swing	24.6 ± 7.2	36.8 ± 8.6	<0.001*	0.97
	Late swing	23.4 ± 5.1	19.8 ± 5.1	0.919	0.46

During preferred speed, the CoA of the motor primitives of the propulsion, early and late swing synergies occurred significantly earlier in MS patients compared to the HP group. (Table 3). That was also true for the weight acceptance synergy during fixed speed. In contrast, at fixed speed the CoA in the early swing synergy occurred significantly later in MS patients compared to the HP group.

**Table 3:** Centre of activity (CoA, average values ± standard deviations in % of gait cycle) of the motor primitives for the healthy participants (HP) and patients with Multiple Sclerosis (MSP) walking at preferred speed (HP = 1.3 ± 0.1 m/s, MSP = 0.8 ± 0.4 m/s) and fixed speed (0.7 m/s).

Speed	Muscle Synergies	HP (%)	MSP (%)	*p-value*	*power*
**Centre of Activity (CoA)**					
Preferred	Weight acceptance	10.3 ± 3.4	10.3 ± 10.7	0.353	0.05
	Propulsion	27.8 ± 2.3	23.7 ± 4.4	0.020*	0.75
	Early swing	75.7 ± 7.5	63.1 ± 8.4	0.004*	0.94
	Late swing	91.6 ± 2.1	87.7 ± 3.2	0.005*	0.90
Fixed	Weight acceptance	12.3 ± 1.8	10.5 ± 0.4	0.005*	0.88
	Propulsion	26.2 ± 1.7	24.7 ± 3.4	0.296	0.31
	Early swing	66.6 ± 4.3	77.0 ± 3.0	<0.001*	0.99
	Late swing	85.8 ± 17.6	87.6 ± 2.2	0.890	0.06

**Figure 5:**
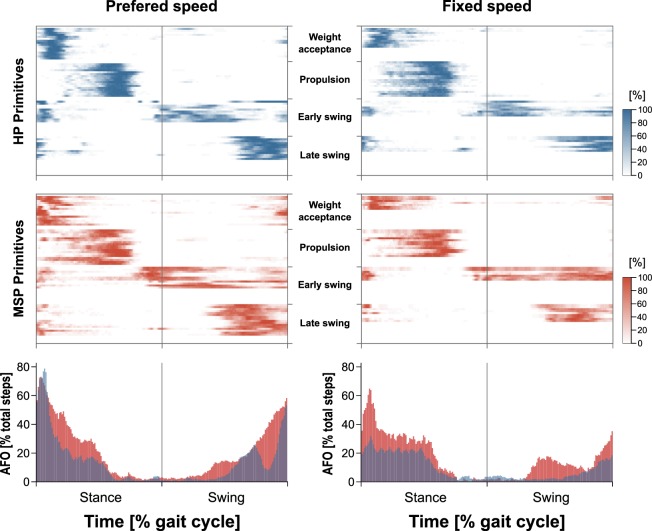
Overlapping time intervals of motor primitives during preferred speed (left) and fixed speed (right). The heat maps show the individual motor primitive when exceeding half maximum activation for healthy participants (HP, blue) and MS patients (MPS, red). Darker colours indicate higher frequencies of occurrence across all 30 gait cycles per participant. At the bottom the averaged frequency of overlaps (AFO) across all gait cycles and all participants per group. For all graphs the x-axis full scale represents one gait cycle (stance and swing normalized to the same amount of points.

In all participants, we identified overlapping motor primitives for both walking conditions during the weight acceptance and early push-off phase as well as during the late swing phase of the gait cycle. The highest number of overlaps was observed during or shortly after touchdown. At preferred speed, the MSP demonstrated a larger number of overlaps of the weight acceptance and push-off motor primitives as well as of the early and late swing motor primitives. These resulted in an increased averaged frequencies of overlaps (AFO) in the respective time phases of the gait cycle for MSP compared to HP (Fig. 5). In contrast the HP showed slightly higher AFOs directly after touchdown. As to be seen from the heat maps, this mainly resulted from the overlap of the motor primitives of the weight acceptance and late swing synergies (Fig. 5). During fixed speed, MSP demonstrated consistently higher AFOs compared to HP that again could be attributed to the overlap of the motor primitives of the two stance phase related and the two swing phase related synergies, respectively.

In the motor modules during walking at preferred speed the MS patients demonstrated significantly higher contributions of the RF (p = 0.042) in the propulsion synergy, the ST (p = 0.008) in the early swing synergy and the RF (p = 0.010), VM (p = 0.048) and VL (p = 0.011) in the late swing synergy compared to the HP group (Fig. 4, left). During walking at fixed speed the MS patients group showed significantly lower relative contributions of the ME (p = 0.003), MA (p < 0.001), RF (p < 0.001), TA (p = 0.013), PL ( < 0.001) and GL (p = 0.002) in the weight acceptance synergy as well as for the RF (p < 0.001) and VL (p < 0.001) in the early swing synergy. In addition, we observed in the MS patients significantly higher contributions of the RF (p = 0.02) in the propulsion synergy as well as for the MA (p < 0.002) and ST (p = 0.016) in the early swing and the MA (p < 0.001), RF (p = 0.004) VL and BF (both p < 0.001) in the late swing synergy compared to the HP (Fig. 4, right).

## Discussion

In the current study, we compared MS patients and age-matched healthy participants in two walking conditions (i.e. preferred and fixed walking velocity) to investigate the modular organization and potential adjustments in motor control. We found higher gait instability (i.e. higher MLE values) in MS patients compared to healthy participants, no significant differences in the amount of muscle synergies between the two investigated groups but alterations in the modular control of walking. MS patients exhibited a widening in the motor primitives (the basic activation patterns), as well as differences in the motor modules (relative contribution of the muscles within the respective synergies). In agreement with earlier reports^9,11,63^ MS patients demonstrated slower gait velocity, shorter step length, lower cadence, longer contact and double stance times compared to HP during walking at preferred speed. The absence of differences in the temporal characteristics between MS patients and HP during walking at fixed speed can be attributed to the very slow velocity of 0.7 m/s used in our study. This argument is in good agreement with the literature^11^ showing difference reductions between MS patients and HP with decreasing walking speeds, in which the lowest walking speed for HP (0.97 m/s^63^) was still higher than in our study.

In human walking, the step-to-step variability results in a local instability of the movement. At the same time this movement is globally stable, if the variations remain within the domain of attraction and the person mange to locomote without falling^64^. In the current study the MLE quantifies the predictability of the walking movements based on the gait cycles and therefore the ability of the system to respond to small perturbations^51,52^. A more chaotic and unstable dynamical system correspond to increased MLE values^57,58^. Increased measures of linear and nonlinear gait variability in MS patients compared to healthy controls have been associated to disturbances in the gait pattern^65^. In our study, the MS patients demonstrated a significantly higher instability of walking at both velocities, represented by ~30% and ~15% higher MLE values in preferred and fixed speed, respectively, compared to HP. In previous studies, MLE increases of ~6% during walking on an uneven surface^20^, ~9% in patients with focal cerebellar lesion^66^ and ~21% in patients with moderate neurological gait disorders^67^ have been observed. The large increase in the MLE values in MS patients compared to HP in our study strongly indicated higher local dynamic instability of MS patients. We propose that the larger local instability in MSP may be associated with the pathology-induced neuromuscular weaknesses when performing the two walking tasks.

Regarding the motor control, four muscle synergies were sufficient to describe the walking task of the lower extremity in MS patients and HP for both, preferred and fixed walking velocity. A similar number of synergies for participants with and without neural disorders was also observed in MS patients in the upper extremity^68^ as well as in the lower extremity during walking in cerebellar ataxic patients^32^ and MSP^33^. The MSP group in our current study was characterized by mild to moderate motor impairment and therefore, it is possible that MS patients with a higher level of impairment might demonstrate changes in the number of muscle synergies. This is supported by some studies suggesting a correlation of the dimensionality of muscle synergies and the level of the neurological motor and functional impairments^69–71^ and a possible relation to merged muscle synergies^72^. The observed lower similarities of the respective motor primitives within the group of MS patients revealed a less consistent temporal structure of motor primitives compared to HP. This reflects larger inter-individual variations in motor control patterns used by the MS patients to accomplish the walking tasks. The four muscle synergies can be attributed to specific gait phases, such as weight acceptance, propulsion, early swing and late swing. The alteration in the basic activation patterns between the two groups was characterized by a widening (i.e. increase in FWHM) in most of the motor primitives in MS patients in both, preferred speed (weight acceptance, early swing, late swing) and fixed walking velocity (weight acceptance, early swing). In some motor primitives this was coupled with significant time shifts to earlier occurring CoA values. During preferred speed this occurred in all motor primitives except the weight acceptance and during fixed speed only in the weight acceptance motor primitive. In contrast the CoA of the early swing motor primitive in MS patients was significantly delayed.

Muscle synergies can be expressed by motor circuits within the cortex, brainstem and spinal cord^26,73,74^. As generally accepted, walking is essentially accomplished by an intrinsic network of spinal interneurons (central pattern generator), generating the rhythm and left-right alternation for locomotion influenced by the CNS^75–78^. The time-dependent fine tuning of elementary spinal commands is assigned to be of a supraspinal origin^73,79–81^. This suggests a modulation of spinal motor modules by the descending commands from the brainstem and motor cortex^29,75^.

The observed widening of the motor primitives in MS patients compared to HP were also present in terms of increased FWHM values in the spinal motor output projected from the EMG signals. Representing prolonged muscle activations, the widening most likely was important to maintain functionality during walking in the MS patients by compensating for pathology-related motor impairments as also described in the literature^33^. Based on findings in sensory impaired animals^82^ and in spinal cord injury patients^83^, the widening of motor primitives can be interpreted as a compensatory mechanism adopted by the CNS to cope with postural instability. Recently, we reported a widening of the motor primitives in HP during walking on an uneven surface^20^. A widening was also observed in HP during walking on slippery ground or on a narrow beam^32^. These findings suggest, that the CNS uses a consistent set of neural control elements with a flexible temporal layout to maintain safe locomotion in the presence of external (e.g. mechanically-induced) perturbations. The assumption is in good agreement with the dynamical system theory approach, where high similarities in behaviours occur because of attractors^84,85^. These attractors can be seen as key factors on different levels that are either necessary or evolved to the best solution to accomplish a certain task. Following this idea, a disturbance of the walking movement would demand for a coordinated motor response to maintain global stability or in other words be able to stay within the same attractor^21^. In our opinion, this response could be widely independent of the origin of the disturbance, such as external or environmental conditions (e.g. uneven surface) or internal sources (e.g. strength of specific muscles, sensory based balance problems, any sensory or motor impairments).

Neuromuscular deficits can introduce internal perturbations during locomotion^21^ and can modify motor behaviour to adequately cope with these disturbances and maintain functionality. In spite of a higher instability, all investigated MS patients were able to perform the walking task in both velocities in the presence of pathology-related neuromuscular deficits. While MS patients demonstrated a reduced preferred speed compared to HP, similar differences in the timing of the motor primitives were also observed at fixed speed, where both participant groups had the same controlled external conditions. Thus, we can assume that functionality was maintained by modifying the shape of the motor primitives and the spinal mapped EMGs. We argue that the observed widening in the motor primitives and spinal motor output mapped from the EMGs in MS patients increase the temporal overlap of muscle synergies and therefore the fuzziness^86,87^ of the temporal boundaries in the modular organization of walking. The increased overlap of the muscle synergies on one hand may represent a reduced ability of MSP to differentially activate specific synergies. On the other hand, this might create a “buffer” of motor control, enhancing the robustness of the motor system to cope with the pathology-related internal perturbations. The coping effort is consistent with findings from Saleh, *et al*.^22^ suggesting locomotion to be more challenging for MS patients compared to HP. Furthermore, our results (i.e. larger variations in the motor primitives in MS patients) indicate that the preservation of the task functionality in presence of neuromuscular deficits was achieved at the expense of accuracy. Compared to HP the reduced accuracy in motor control of the MS patients occurred in timing and duration of the motor primitives related to mechanical function within the gait cycle as well as in the lower similarity of motor control and movement solutions for the walking task. In addition to the widening of motor primitives, the MS patients demonstrated redistributed motor modules. In particular, the redistribution of muscle contribution of the weight acceptance synergy during fixed speed indicated a higher importance of the medio-lateral postural stability. In the motor modules this was represented by the dominant FL muscle while the relative contribution of the MA, RF, TA, PL and GL in MS patients were significantly reduced compared to HP.

In summary, MS patients revealed greater instability and modified the modular organization of the neuromuscular control during walking compared to healthy controls in order to cope with the pathology-related internal perturbations. Although the same overall number of muscle synergies for both participant groups was sufficient to describe the motor pattern of walking at preferred and slower fixed velocity, we identified a widening of the basic temporal activation patterns (motor primitives and EMG-based spinal motor output) that, partly coupled with time shifts, resulted in an increased overlap (fuzziness) of the muscle synergies. The alterations in the temporal structure sometimes were coupled with redistributions of the relative muscle contribution within the respective synergies (motor modules). We propose that the increased fuzziness of the temporal boundaries in the modular organization of walking create a “buffer” of motor control increasing its robustness. This helps MS patients to maintain functional gait patterns.

It must be taken into account, that this study has some limitations. First, the relatively small number of participants of older adults only allows for a limited direct generalisation of the results. However, for the healthy participants the current results closely correspond to our previous work^37,88^ and to other existing literature reports^30,31^. Second, the current results were generated only during treadmill walking at preferred and a fixed speed. Future work might include overground walking as well as specified challenging conditions for MS patients to better evaluate possible compensatory mechanisms compared to a participant specific baseline dataset.
